# First-Principles Study of Rh Segregation in the Au–Rh(111) Alloy with Adsorbed NO, CO, or O_2_

**DOI:** 10.3390/molecules30112389

**Published:** 2025-05-30

**Authors:** Yufeng Wen, Yanlin Yu, Huaizhang Gu, Yuexin Kang, Guoqi Zhao, Yuanxun Li, Qiuling Huang

**Affiliations:** 1 Key Laboratory of Advanced Functional Materials, School of Science, Kaili University, Kaili 556011, China; kluwenyf@163.com (Y.W.); guhzh666@163.com (H.G.); kangyuexin2025@163.com (Y.K.); zhaoguoqi1986@163.com (G.Z.); lyxlyy060602@126.com (Y.L.); 13312344265@163.com (Q.H.); 2 School of Mathematics and Physics, Jinggangshan University, Ji’an 343009, China

**Keywords:** first-principles calculation, adsorbate-induced surface segregation, Au–Rh(111) alloy

## Abstract

Adsorbate-induced surface segregation significantly influences the catalytic and electrochemical performance of bimetallic alloys. Using density functional theory (DFT), we investigated Rh segregation in Au–Rh(111) alloys under the influence of adsorbed NO, CO, or O_2_. The computational results reveal that these adsorbates can markedly alter Rh segregation trends on the Au–Rh(111) surface. Under vacuum conditions, the Rh atom remains preferentially in the bulk of the alloy; whereas, in the presence of adsorption, it segregates to the topmost layer, where NO has the greatest influence, followed by CO and O_2_. Electronic structure analysis and adsorption energy evaluations further reveal that the strength of the surface–adsorbate interactions critically governs the Rh segregation behavior under reactive conditions. These findings establish a theoretical framework for designing Au–Rh alloys as efficient catalysts for CO oxidation.

## 1. Introduction

Bimetallic alloys are recognized for their potential to enhance the performance of catalysts through synergistic effects, making them a focal point of research in catalysis [1,2]. Consequently, they have attracted significant interest in both industrial applications and fundamental studies [3,4]. The catalytic efficacy of an alloy surface is strongly influenced by its composition and structural features. These surface characteristics frequently differ from the bulk material’s properties, primarily because of elemental segregation within the alloy [5,6]. Especially in a reactive environment, the composition and structure of an alloy surface may further change if one of the alloying elements interacts strongly with adsorbed molecules, causing adsorbate-induced segregation [7,8]. A wide range of experimental and computational research has revealed that the adsorbate-induced segregation could occur across a variety of bimetallic systems, exerting a profound influence on their catalytic behavior [9,10,11,12,13]. For instance, Wang et al. [11] reported that chemisorbed oxygen prompted Mo segregation in Ni-based hydrogen evolution electrodes, which in turn reduced the reaction rate. Gasteiger et al. [12] showed that the segregation of Co induced by O_2_ reduced the catalytic efficiency of Pt–Co catalysts in the oxygen reduction reaction. Similarly, McCrory et al. [13] observed that OH adsorption on the surface of Ni–Fe catalysts led to the segregation of Fe, significantly decreasing the oxygen evolution reaction activity. Therefore, it is critical to understand whether a bimetallic system with targeted properties remains stable under the environmental conditions of its intended application.

Among bimetallic systems, Au alloys are recognized as effective catalysts for reactions, such as hydrogen oxidation reaction [14], dehydrogenation reaction [15], hydrogen evolution reaction [16], hydrogenation reaction [17], oxygen reduction reaction [18], reforming reaction [19], water–gas shift reaction [20], and so forth. In the context of CO oxidation, many studies have demonstrated that Au alloys with Rh could enhance the catalytic activity for CO oxidation and further improve the stability of the catalytic reaction [21,22]. This is because Rh atoms could promote the formation of an Au-segregated surface structure with higher catalytic activity, which is conducive to the enhancement of catalytic stability for CO oxidation [22]. In addition, the results obtained by Mancilla et al. [21] also demonstrated that Au alloys with Rh tended to form Au-segregated surface structures, which not only exhibited superior catalytic activity, but prevented the oxidation of Rh during CO oxidation. These exceptional catalytic properties have also led to the use of Au–Rh alloys in automotive catalytic converters to improve efficiency and reduce emissions. However, in this practical application, the alloy catalysts are inevitably exposed to a variety of reactive gases, including NO, CO, and O_2_ [23]. The presence of these reaction gases may induce Rh segregation on the catalyst surface, which could significantly decrease the catalytic activity of the Au–Rh catalysts in the CO oxidation, thus affecting the efficiency of automotive catalytic converters [24]. However, there are few experimental and theoretical studies discussing which reactive gases lead to the experimentally observed segregation of Rh on alloy surfaces. Obviously, comprehensive theoretical calculations are essential for gaining a deeper understanding of the experimental observations.

In this study, we employ first-principles calculations to examine how chemisorbed NO, CO, or O_2_ influence the segregation tendencies of Rh atoms within the Au–Rh(111) alloy surfaces. The results reveal that these adsorbates significantly affect Rh segregation on the Au–Rh(111) surface. Under vacuum conditions, the Rh atom remains preferentially in the bulk of the alloy; whereas, in the presence of adsorption, it segregates to the topmost layer, where NO has the greatest influence, followed by CO and O_2_. Electronic structure analysis and adsorption energy evaluations further reveal that the strength of the surface–adsorbate interactions critically governs the Rh segregation behavior under reactive conditions. The remainder of this paper is structured as follows: Section 2 outlines the theoretical methods and computational details, Section 3 presents the results and discussion, and Section 4 provides a brief summary.

## 2. Materials and Methods

The spin-polarized DFT calculations were performed employing the Vienna Ab initio Simulation Program (VASP) [25,26,27] with the Perdew–Burke–Ernzerhof (PBE) functional [28] as exchange–correlation functional to obtain the energy. All calculations were conducted on the (111) crystallographic planes, which are recognized as the most thermodynamically stable configuration for face-centered cubic (fcc) metals. For core electron interactions, the projector augmented wave (PAW) approach was utilized [29,30]. A plane-wave cutoff energy of 400 eV was selected after optimization. To calculate electron occupancies, the Methfessel–Paxton method [31] with a width of the smearing in 0.2 eV was applied. The calculations are carried out using the 3 × 3 × 1 Monkhorst–Pack [32] mesh k-points for surface calculations, and the electric dipole was neglected. The converge criteria for force and electronic self-consistency were 0.02 eV/Å and 10^−5^ eV, respectively.

**Figure 1:**
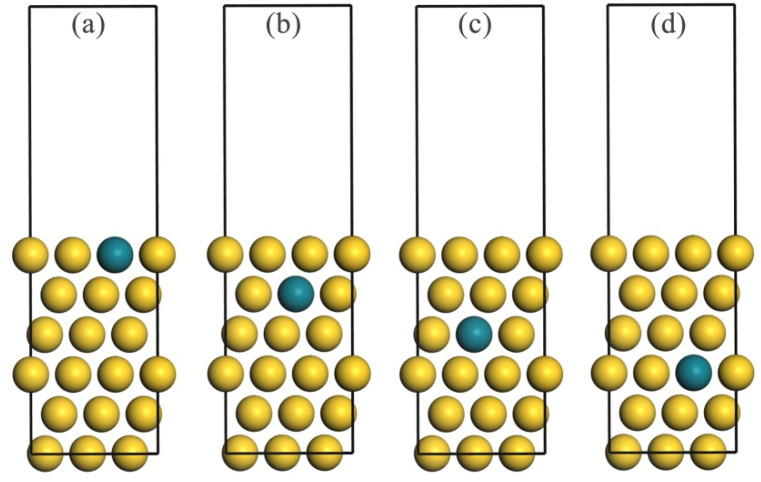
The Au–Rh(111) alloy slab model showing substitution of an Au atom with an Rh atom in the (**a**) first, (**b**) second, (**c**) third, and (**d**) fourth atomic layer. Gold and green spheres correspond to Au and Rh atoms, respectively.

The DFT-calculated lattice constant for bulk Au is 4.16 Å, which is consistent with previously reported values [33,34,35]. The pure copper slab model included six atomic layers representing a 3 × 3 supercell, separated by 15 Å of vacuum space. This choice was based on prior studies of Au-based bimetallic systems [33,36,37], where the six–seven atomic layer was shown to balance computational cost and accuracy in surface energy and adsorption property calculations. In the Au–Rh alloy system, one Rh atom replaced one Au atom in each layer of the pure Au slab model, as illustrated in Figure 1. To simulate both bulk and surface environments, the bottom two layers of the slab were fixed, while the top four layers, along with the adsorbed reactive gases, were allowed to relax.

The surface segregation energy ($E_{segr}$) is defined as the energy difference between configurations where the Rh atom occupies the upper surface layer versus the bulk [33,38]. Therefore, this parameter is computed using Equation (1) as follows:

(1) $$E_{segr}=E_{Au\text{–}Rh\left(Rh,x\text{-}layer\right)}-E_{Au\text{–}Rh\left(Rh,4th\text{-}layer\right)}.$$

In this equation, $E_{Au\text{–}Rh\left(Rh,x\text{-}layer\right)}$ and $E_{Au\text{–}Rh\left(Rh,4th\text{-}layer\right)}$ denote the total energies of the Au–Rh alloy system. Here, *Au–Rh*(*Rh, x-layer*) corresponds to configurations where the Rh atom is positioned within the upper *x*-th Au atomic layer (*x* = 1, 2, or 3), while *Au–Rh*(*Rh, 4th-layer*) refers to the configuration where the Rh atom is positioned within the fourth Au layer, which is analogous to the “bulk” Au matrix. Equation (1) above indicates that the higher value of $E_{segr}$ correspond to the increased difficulty in achieving surface segregation.

The adsorption energies ($E_{ads}$) of molecular CO, O_2_, and NO adsorbed on the pure Au and Au–Rh(111) surfaces were computed using the following approach:

(2) $$E_{ads}=E_{gas/surf}-E_{surf}-E_{free\text{-}gas}.$$

In this equation, $E_{gas/surf}$ refers to the total energy of the adsorbed system, $E_{surf}$ denotes the bare surface energy, and $E_{free\text{-}gas}$ represents the energy contribution from the reactive gas phase.

The d-band center, which quantifies the change in the d-states of the alloy component, is given by the following equation:

(3) $$\varepsilon_{d}=\frac{\int_{-\infty }^{+\infty }E\rho \left(E\right)dE}{\int_{-\infty }^{+\infty }\rho \left(E\right)dE}$$

In this equation, *E* denotes the specified energy, while *ρ*(*E*) represents the electronic state density.

## 3. Results and Discussion

### 3.1. Adsorption Behavior

Given that both Au and Rh are metals with face-centered cubic structures, it is observed that CO, NO, and O_2_ molecules exhibit a preference for adsorption on the (111) surface of these metals [33,39]. For both the CO and NO molecules, four primary stable adsorption sites are identified on this surface: Top, Bridge, fcc, and hcp. For O_2_ molecules, the stable adsorption sites primarily comprise top–bridge–top (bridge), top–hcp–bridge (hcp), and top–fcc–bridge (fcc).

Table 1 summarizes the adsorption energies for the studied gas molecules on both Au(111) and Rh(111) surfaces. For the Au(111) surface, CO exhibits the strongest adsorption, surpassing NO and O_2_. Both CO and NO adopt a linear binding geometry, where the carbon atom of CO and the nitrogen atom of NO orient toward the surface. These molecules also share identical adsorption site preferences, specifically occupying the top sites on the (111) surface. Additionally, in accordance with the findings of the previous research [40,41], the Au(111) surface does not readily support the chemisorption of O_2_ unless external energy inputs, such as electron bombardment or UV irradiation, provide the necessary activation energy.

For the Rh(111) surface, it was observed that the adsorption of CO is stronger than that of O_2_, but remains weaker than that of NO. Both CO and NO still adopt a linear binding geometry, and these molecules demonstrate a uniform adsorption site preference, exhibiting a marked predilection for occupying the fcc or hcp sites on the (111) surface. Additionally, in comparison with Au, Rh exhibits a higher affinity toward reactive gaseous molecules. For instance, the lowest adsorption energies for CO and NO on the Rh(111) surface were found to be −1.86 and −2.37 eV, respectively, whereas the relevant values for the same molecules on the Au(111) surface were −0.35 and −0.28 eV, respectively.

The adsorption configurations of gas molecules on the Au–Rh(111) surface have been systematically investigated, and the lowest adsorption energies of gas molecules on the alloy surface have been determined by calculations for different positions of the Rh atoms, as shown in Table 2. The calculated results reveal that, when the Rh atom is situated in the topmost layer of the Au–Rh(111) surface, the order of the adsorption strength is NO > CO > O_2_, and both NO and CO preferentially bind at the top site of Rh atom. Nevertheless, the most stable adsorption configuration of O_2_ on the alloy surface is top–fcc–bridge, where one oxygen atom occupies the top site of Rh and the other occupies the bridge site formed by two Au atoms. When the Rh atom migrates into the second layer of the Au–Rh(111) surface, the adsorption of O_2_ on the alloy surface is not stable, and both NO and CO preferentially occupy the top of the Au atom in close proximity to the Rh atom. Nevertheless, their adsorption strength is considerably diminished. Notably, when the Rh atom migrates into bulk regions (third and fourth layers), The adsorption behavior of the three gases on the alloy surface is very similar to that on the surface of Au(111).

### 3.2. Segregation Behavior

**Figure 2:**
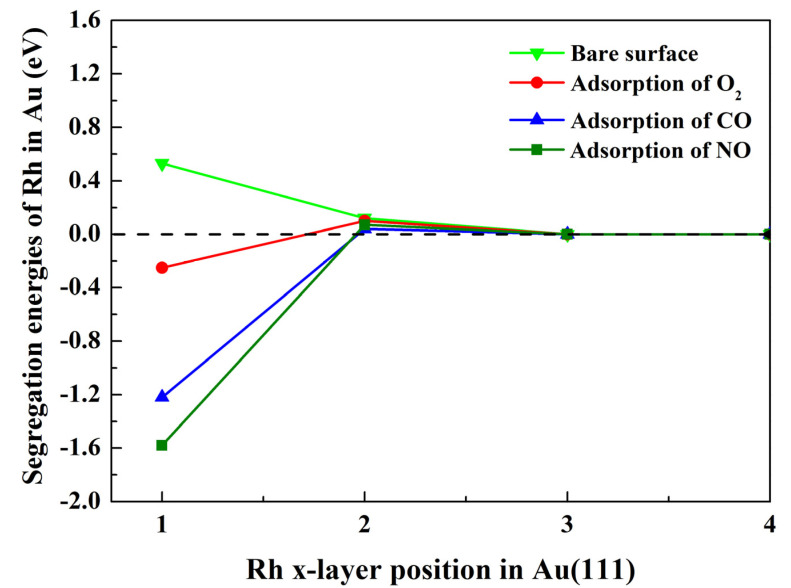
Evolution of the segregation energies (eV) of Rh from the Au ‘bulk’ (4th layer) to the upper surface layers with and without adsorbed NO, CO, and O_2_ molecules.

The segregation energies of Rh in Au–Rh(111) alloys were computed with and without adsorbed NO, CO, and O_2_ molecules, as illustrated in Figure 2. For the clean surface, i.e., without adsorbed gas molecules, Rh exhibits a positive segregation energy of 0.53 eV in the first atomic layer, indicating a thermodynamic preference to remain within the bulk. Surface segregation in metallic alloys is fundamentally driven by the minimization of the system’s Gibbs free energy, where two dominant factors govern this process under vacuum conditions, including (1) the relative surface energies of constituent elements, and (2) the elastic strain energy relaxation [42,43]. While the entropy-driven randomization of the atomic distributions becomes significant at elevated temperatures, its contribution is inherently excluded in our T = 0 K DFT framework. For clean surfaces, elements with higher surface energies and smaller atomic radii tend to resist segregation to the surface [6,38]. These principles explain Rh’s bulk preference in Au–Rh alloys: Rh exhibits a surface energy nearly double that of Au (2.7 vs. 1.5 eV/atom [44]) and a smaller atomic radius (2.34 Å vs. 2.43 Å for Au [45]), creating both thermodynamic and steric barriers to surface migration. This interpretation aligns with prior computational studies showing limited Au segregation in Rh-rich matrices under vacuum [46,47], confirming that surface energy disparities and atomic size effects dominate segregation behavior in the absence of adsorbates. When Rh is situated in the second layer of the alloy, the segregation energy decreases significantly, with a value of only 0.12 eV. Notably, when Rh is situated in the third layer of the alloy, the value of the segregation energy is close to zero, suggesting this layer can be considered to be a bulk layer like the fourth layer. Analogous behavior is observed under gas adsorption conditions.

In the case of the adsorption of gas molecules, when Rh is situated in the first atomic layer, the segregation energy is reversed to negative values. These were found to be −1.58 eV, −1.22 eV, and −0.25 eV, for NO, CO, and O_2_, respectively. Negative segregation energies indicate a thermodynamic preference for Rh to segregate to the Au–Rh(111) surface under adsorbate influence, and the order of influence on Rh segregation is NO > CO > O_2_. This phenomenon aligns with the Le Chatelier principle: adsorbate-induced surface chemical potential gradients drive segregation to stabilize the system [48]. In fact, some adsorbate-induced negative segregation has been validated in Au–Rh systems. For example, DFT calculations by Sansa et al. [37] predicted Rh segregation in the Au–Rh(111) surface under CO adsorption, closely matching our calculated results. The experimental studies by Mancilla et al. [21] and Wang et al. [22] demonstrated CO-induced Rh segregation in Au–Rh alloys, consistent with our DFT predictions for CO–Au–Rh systems. According to the findings of previous studies [8,49], adsorbate-induced segregation is determined by a combination of three factors. These factors comprise the surface energy of the alloying element, the atomic radius of the alloying element, and the adsorption strength between the adsorbed gas and the alloy surface. There are two offsetting factors that limit this segregation trend. One is that the surface energy of Rh is higher than that of Au [44], and the other is that the atomic radius of Rh is smaller than that of Au [45]. In addition, the standard DFT calculations are performed at 0 K and do not explicitly include temperature effects. However, even if the temperature effect is not taken into account, it does not affect the tendency of Rh to segregate. This is because, for strong adsorbates, the chemisorption effect dominates over thermal energy. Therefore, this change in the trend of segregation is attributed to the stronger binding of NO, CO, and O_2_ to the alloy surface.

### 3.3. Electronic Structure Analysis

**Figure 3:**
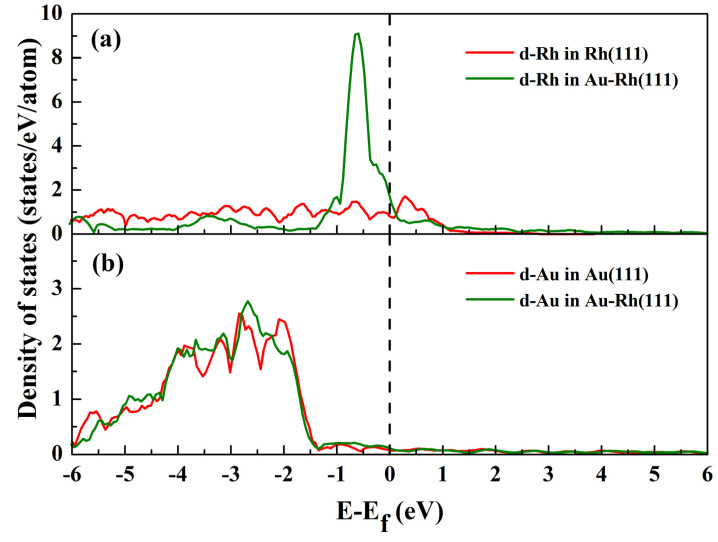
Calculated density of states (DOS) for Rh and Au atoms in the alloy and pure metal surfaces. (**a**,**b**) present the d-band DOS for Rh and Au atoms in Au–Rh(111) surfaces, respectively. The d-band DOS for pure metal surfaces is presented for comparison.

To evaluate the local effect of Rh alloying on the electronic characteristics of the Au–Rh(111) surface, the d-band DOS for Rh and Au atoms in the outermost surface layer under vacuum conditions was calculated, as illustrated in Figure 3. Relative to pure Rh, Rh within the alloy displays a narrowed d-band DOS shifted toward higher energy levels near the Fermi energy (Figure 3a). This finding implies electron donation from Rh to adjacent Au atoms, which results in an increase in d-band electrons in Au. This will cause the d-band center for Au to shift down, away from the Fermi energy level. This phenomenon is consistent with the results shown in Figure 3b. According to the d-band center model developed by Hammer and Nørskov [50,51], these shifts of the d-band centers, as shown in Figure 3, are closely related to the gas adsorption strength, which will drive the segregation of Rh on the surface of the alloy.

**Figure 4:**
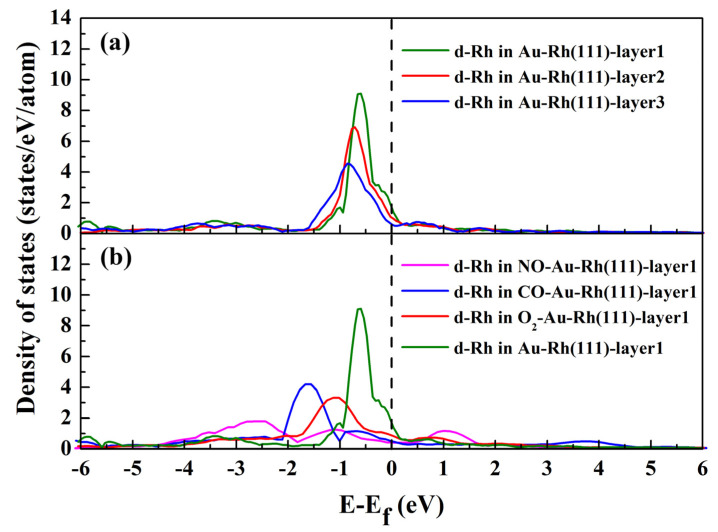
Calculated d-band DOS for the Rh atom located in three upper layers of Au–Rh(111) surface without adsorption (**a**), and in the first layer of Au–Rh(111) surface with adsorption (**b**), respectively.

To further explore the preferential location of the Rh atom within the Au ‘bulk’ without adsorption and the surface segregation with adsorption, we evaluated the d-band DOS for the Rh atom located in three upper layers of the Au–Rh(111) surface without adsorption (Figure 4a), and in the first layer of the Au–Rh(111) surface with adsorption (Figure 4b), respectively. As shown in Figure 4a, the shape and position of the Rh atom’s d-band DOS depend critically on its position within the surface layers. When Rh migrates from the third to the outermost atomic layer, its d-band DOS progressively shifts to higher energy levels and narrows in width. This demonstrates that electronic interactions under vacuum conditions govern Rh’s preferential occupancy within the Au ‘bulk’ structure [33]. As shown in Figure 4b, the d-band DOS for surface Rh atoms in the alloy exhibits significant broadening under adsorbate exposure, particularly with NO adsorption. This narrowing in DOS under non-adsorbed conditions arises from the reduced coordination of surface atoms relative to bulk atoms. In adsorbed systems, the presence of adsorbates enhances surface atom coordination, leading to a broadening of the DOS [52]. Additionally, the broadened DOS shifts toward lower energy levels under adsorption conditions. Such a redistribution likely lowers the system’s total energy, thereby enhancing its thermodynamic stability.

To probe how adsorbates modulate the electronic structure of the Au–Rh(111) surface, we performed Bader charge analysis on the top-layer Rh atoms under both clean and adsorbate-covered conditions. In the absence of adsorbates, the Au atoms gained 0.1 electrons from their neighboring Rh atom, demonstrating significant charge transfer that stabilizes Ni within the bulk through covalent Cu–Ni bonding. However, adsorbate interactions dramatically alter this charge balance: when NO, CO, and O_2_ adsorb onto the surface, they gained 0.14, 0.15, and 0.18 electrons from Rh atom, respectively. This electron depletion weakens the covalent Au–Rh bonds that originally anchored Rh in the bulk, creating a thermodynamic driving force for Rh segregation toward the surface to compensate for the charge loss.

## 4. Conclusions

First-principle computational methods were employed to investigate Rh segregation in Au–Rh(111) alloys under the influence of adsorbed NO, CO, or O_2_. Our findings reveal that these adsorbates can markedly alter Rh segregation trends on the Au–Rh(111) surface. Under vacuum conditions, Rh atom remains preferentially in the bulk of the alloy; whereas, in the presence of adsorption, it segregates to the topmost layer, where NO has the greatest influence, followed by CO and O_2_. Examining the adsorption behavior of these adsorbates on Au–Rh(111) demonstrates a binding strength order of NO > CO > O_2_, with all three adsorbates exhibiting stronger affinity for Rh than Au. An electronic structure analysis for the clean Au–Rh(111) surface shows that electron donation from Rh to the adjacent Au atoms results in an increase in d-band electrons in Au. This will cause the d-band center for Au to shift down, away from the Fermi energy level. The shape and position of Rh’s d-band DOS demonstrates that electronic interactions under vacuum conditions govern Rh’s preferential occupancy within the Au ‘bulk’ structure. Notably, in the presence of adsorption, the d-band DOS broadens and shifts to lower energies, potentially lowering the system’s total energy and enhancing stability. These insights offer a theoretical foundation for leveraging Au–Rh alloys in CO oxidation catalysis.

## Figures and Tables

**Table 1:** Adsorption energies (eV) for NO, CO, and O_2_ molecules on both Au(111) and Rh(111) surfaces. Configurations with the lowest energy are highlighted in bold.

		Top	Bridge	fcc	hcp
Au(111)	NO	**−0.28**	−0.18	−0.10	−0.07
	CO	**−0.35**	−0.26	−0.18	−0.17
	O_2_	No adsorption			
Rh(111)	NO	−1.85	−2.17	**−2.37**	−2.37
	CO	−1.80	−1.79	−1.85	**−1.86**
	O_2_	No adsorption	−1.28	**−1.32**	−1.31

**Table 2:** The adsorption energies (eV) for NO, CO, and O_2_ molecules on the Au–Rh(111) surface for different positions of the Rh atom.

Position of the Rh Atom	NO	CO	O_2_
1st layer	−2.44	−2.14	−0.79
2nd layer	−0.37	−0.46	No adsorption
3rd layer	−0.31	−0.37	No adsorption
4th layer	−0.31	−0.38	No adsorption

## Data Availability

The authors confirm that the data supporting the findings of this study are available within the article.
